# Advancing the use of artificial intelligence in health technology assessment activities: insights and next steps from the 2025 HTAi Global Policy Forum

**DOI:** 10.1017/S0266462325103395

**Published:** 2026-01-08

**Authors:** Rebecca Trowman, Meindert Boysen, Antonio Migliore, George Valiotis

**Affiliations:** Health Technology Assessment International, Canada

**Keywords:** generative artificial intelligence, large language models, health technology assessment, trust, human agency, AI integration, healthcare systems, data privacy, HTAi, policy, ethics, bias

## Abstract

The use of Artificial Intelligence (AI) in Health Technology Assessment (HTA) activities presents an opportunity to enhance the efficiency, accuracy, and speed of HTA processes worldwide. However, the adoption of AI tools in HTA comes with diverse challenges and concerns that must be carefully managed to ensure their responsible, ethical, and effective deployment. The 2025 Health Technology Assessment international Global Policy Forum (GPF) informed GPF members of the integration of AI into HTA activities, with a particular focus on the use of Generative AI (GenAI). With the overarching goal of illuminating and inspiring tangible outputs and actionable recommendations, the event brought together a diverse range of interest holders to explore the opportunities and challenges of AI in HTA. This article summarizes the key discussions and themes that informed the GPF outcomes, including trust, human agency, and risk-based approaches, culminating in a proposed set of priority next steps for the HTA community regarding the integration of GenAI. It also highlights insights into the current state of digital transformation within HTA organizations and the life sciences industry, providing insights into where the field stands and where it is heading.

## Introduction

The pace of technological development, rising healthcare costs, and increasing inequalities are placing pressure on global health systems, and there is a pressing need for these systems to adapt (1). Artificial Intelligence (AI) provides potential to improve system efficiency (in terms of both speed and accuracy) on a global scale. However, the integration of AI into Health Technology Assessment (HTA) activities presents both opportunities and challenges (2). Of particular interest are Generative AI (GenAI) tools, which are defined as “a type of AI that can generate human-like text and creative content (e.g., music and images), as well as consolidate data from different sources for analysis” (3). Using GenAI tools could enhance data processing and synthesis, assist in preparing for decision-making, and bring predictive capabilities with potentially far-reaching implications for the HTA community (4). The life sciences and medical technology industries are already actively participating in the exploration of GenAI for the generation of clinical evidence, and are beginning to use GenAI tools in the HTA submission process (e.g., in developing and adapting HTA dossiers). HTA bodies are therefore striving to assess health technologies more effectively and efficiently, but with increasing resource constraints (4;5).

However, the adoption of GenAI into HTA activities is a complex issue, primarily due to concerns about the trust associated with the use of GenAI tools, their transparency, and the ethical use of the technology (6). Effective GenAI governance and regulation are crucial for ensuring equitable access to GenAI tools and reducing fragmented use across the global HTA ecosystem (7). The 2025 Health Technology Assessment international (HTAi) Global Policy Forum (GPF) was convened to discuss the use of AI in the conduct of HTA activities. The main aims of the HTAi GPF were, therefore, to inform, illuminate, and inspire GPF members on the use of AI in HTA life cycle activities, with the overall goal to develop recommendations for tangible outcomes and next steps related to the topic. These governance measures should align with emerging frameworks, such as the European Union (EU) AI Act (8), World Health Organization ethical AI guidelines (9), and national HTA policies, ensuring coordinated implementation across regions.

Insights were gathered from the HTAi GPF membership and HTAi Interest Groups (IGs) before the GPF through a survey and a pre-GPF webinar. These helped inform the background paper that was developed as pre-reading for the GPF (10). The background paper highlighted mixed experiences and feelings toward the adoption of AI in the conduct of HTA activities. This multitude of views was particularly apparent when considering GenAI. The GPF member survey noted a range of beliefs that could arise from the use of GenAI, including potential increased efficiency (i.e., speed and accuracy of HTA), cost savings, and improved transparency. GPF members felt that the future of HTA – if the power of GenAI is harnessed responsibly – could free up humans in the HTA process to employ more strategic thinking and action while employing their deeper reasoning capabilities, potentially enabling “living” or “real-time” HTA (11). However, these beliefs were also countered by multiple responses highlighting issues around trust, transparency, and resourcing requirements for implementing GenAI, as well as concerns about how this could be achieved practically and in a safe and responsible manner.

## Meeting structure and format

On 26–28 January 2025, 94 delegates met in Cascais, Portugal, for the annual HTAi GPF. Members represented an equal number of not-for-profit organizations (HTA agencies, payers, and health systems) and for-profit organizations (pharmaceutical, biotech, and device companies). Patient representatives, invited speakers, and HTAi leadership also actively participated during the event. The sessions and content were organized as described in Table 1.

**Table 1.** Format of the Health Technology Assessment international (HTAi) Global Policy Forum 2025

Aim	Session(s)	Objectives
Informing	*Pre-GPF Webinar and Day 1*: Background presentations and panel session	Setting the scene: Exploring the possibilities of AI in HTA
Illuminating	*Day 2*: Case studies and policy perspective in an “Open Exchange” and debate	Advancing AI in HTA: Challenges, opportunities, and ethical considerations
Inspiring	*Days 2 & 3*: Themed breakout group discussions and report backs	Defining the next steps

Abbreviations: GPF, Global Policy Forum; AI, artificial intelligence; HTA, health technology assessment.

On Day 1, the GPF provided a broad overview of AI, and then a closer look at the potential GenAI may have across healthcare systems, followed by an in-depth analysis of its current and future applications within HTA activities. Presentations and panel discussions focused on the evolving role in the use of GenAI tools in HTA activities and the diverse views of HTA community members on the issue. Day 2 focused on the practical challenges and ethical considerations of integrating GenAI into HTA processes. Presentations provided case studies and insights into the operational, regulatory, and ethical dimensions of the use of GenAI in HTA activities and beyond.

**Figure 1.**
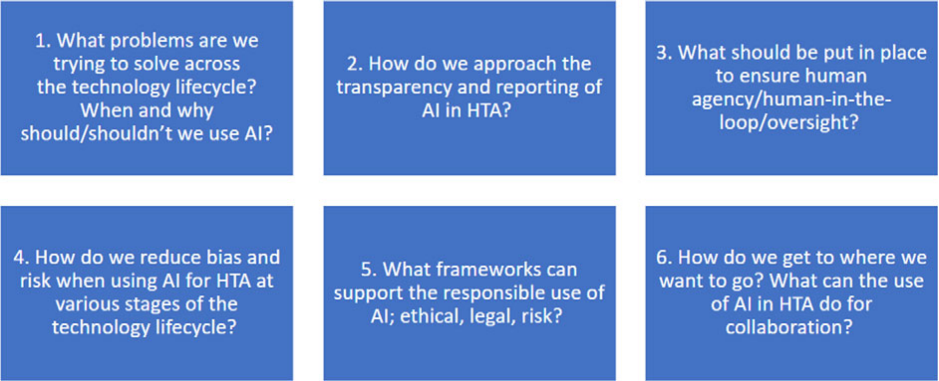
Themes for the breakout groups. Legend: AI, artificial intelligence; HTA, health technology assessment.

On days 2 and 3, six smaller discussion groups were created among the GPF membership, and each group had an assigned facilitator and rapporteur. The breakout groups remained the same for the two breakout sessions on days 2 and 3 to enable deeper conversations into the theme they had been assigned. The themes for the breakout groups are described in Figure 1.

### Meeting discussions

The atmosphere at the HTAi 2025 GPF was charged with optimism and a shared enthusiasm to move forward collaboratively, with the notion that the time to act is now. However, there was alignment in the fact that we must proceed cautiously and not diminish scientific rigor in doing so. GPF members highlighted the importance of working together, leveraging existing efforts, and avoiding the duplication of work that had already been initiated in various sectors. In particular, the life sciences industry could contribute to this roadmap by actively participating in standards development and sharing insights from early GenAI implementation.

The GPF began by considering how the use of AI in HTA activities has shifted from sporadic to exploratory. Before the relatively widespread accessibility of GenAI around 2023, the use of AI in HTA was more limited and targeted to very specific activities. However, as access to more powerful models has increased globally and there has been a shift from dedicated single-purpose models to general-purpose foundational models, AI has started to play a greater part in HTA activities (12). The result of this is that there is no need to train AI models for each application, with “off-the-shelf” models capable of being used for a broader range of applications, generating savings in terms of time, money, and data requirements. At present, these applications appear to be mainly in the context of Health Economics and Outcomes Research, where the use of GenAI by the life sciences industry has been most extensively explored to date (13;14). For example, the use of GenAI for generating search strings, screening abstracts, extracting data, conducting network meta-analyses, and developing and/or adapting economic models is considered the most common use cases in HTA to date (15–17). The acceptability and use of GenAI within, and by, individual HTA bodies is variable, with some organizations taking a very cautious approach with no internal access to GenAI tools, through to early-stage experimentation with existing AI tools and the development of internal, or externally sourced, AI tools and policy statements on the use of GenAI in HTA activities (18–21).

The HTAi GPF discussion highlighted the need for clear guidelines relevant to all system interest holders on the ethical deployment of GenAI tools in HTA activities to enable alignment and a mutual understanding of acceptance across jurisdictions and by interest-holder type. Trust, transparency, and data privacy (including where and how data are stored) emerged as recurring themes of concern that should be proactively and dynamically addressed. Participants discussed the challenges of ensuring content generated by GenAI tools can be distinguished from human work, with the importance of disclosing the use and choice of GenAI tools, and the benefits of using open-source GenAI tools, highlighted. This is akin to current methods, where the databases searched, software used, and traceable flowcharts for each step of a systematic review would be presented. It would be valuable for the HTA community to develop a working definition of levels of human oversight, also known as “human-in-the-loop,” recognizing that acceptability and interpretations may differ across jurisdictions and according to the levels of risk involved. For example, the need to verify GenAI outputs, with human oversight, was considered critical to ensure that GenAI outputs are reliable and valid. Additionally, the importance of addressing data security (including where data are stored and user awareness of this) and minimizing bias in AI models was emphasized. The HTAi GPF reinforced the need for ethical and responsible GenAI adoption in HTA activities, including the involvement of patients and clinicians to establish appropriate use cases for GenAI tools. Collaboration with all relevant interest holders, including the life sciences industry, payers, and regulatory bodies, and upskilling the HTA community were also emphasized as vital components toward the digital transformation of HTA.

Presentations also included results from a survey of European patient groups (22), which indicated strong support for the potential of GenAI to enhance healthcare quality, and this was discussed in relation to the impact on HTA. However, concerns about data privacy, algorithmic bias, and transparency remained prominent. Principles to ensure the ethical deployment of GenAI are needed to preserve human dignity and respect, address data quality and safeguard privacy, foster patient and clinician involvement to co-create solutions, maintain human oversight in decision-making, and prioritize digital literacy and education. This includes providing users with clarity over where data are stored and how data are used in the future when they are entered into AI tools, and how this might potentially infringe copyright, impact intellectual property rights, and compromise confidential data sharing.

Representatives from for-profit organizations (i.e., the life sciences industry) highlighted where GenAI is already being used across the different stages of the technology development life cycle, from molecule identification to drug/device development through to regulatory documentation (including language translation). This was expanded upon with discussion about the European Medicines Agency and the European Medicines Regulatory Network workplan (23;24) to improve efficiency and guide AI tools, especially in preparation for the EU AI Act, which will be Europe’s legal framework that will guide the implementation and uptake of AI in Europe (25).

New academic projects were discussed with a focus on the recently completed HTx (26) and the newly established SUSTAIN-HTA project (27). The latter has been introduced as a European-funded initiative to bridge the gap between academia and HTA bodies, aiming to align HTA needs with new methodological developments in the field, including those pertaining to the use and evaluation of AI more broadly and GenAI. Further detail on whether industry-led GenAI initiatives are coordinated with HTA bodies and/or academia or pursued independently could help assess alignment with HTA processes.

### Key themes

Based on these discussions and insights gathered during the GPF, several critical themes emerged as foundational to advancing the use of AI, particularly GenAI, in HTA activities. These themes are reported below and should not be viewed as sequential steps that need to be taken, but as overarching considerations and efforts that are required in the responsible implementation of AI.

### Trust in AI

Building trust in GenAI tools was recognized as critical, and participants agreed on the importance of increasing transparency throughout the process to ensure the responsible use of GenAI tools in HTA activities (28). Participants recognized that the potential of GenAI in HTA will be contingent on the degree to which trust can be built with interest holders, including healthcare providers, policymakers, patients, and the general public. Ways to build key elements of trust identified included transparency, human accountability, and rigorous testing and validation processes to demonstrate the reliability and robustness of GenAI tools for the various use cases to which they are applied. The ability to share GenAI tools in an open-source manner was seen as one important way of establishing and embedding trust in the tools used (29). Developing gold-standard benchmarks for specific tasks, which can be used to demonstrate the performance of individual tools, would also help build trust, particularly if open-source tools are not available/feasible.

### Human agency in AI implementation

There was alignment that while AI (particularly GenAI) has the potential to automate many tasks within HTA, human involvement is still essential throughout, although this level of involvement may change as GenAI tools evolve and trust is built (30). Maintaining human agency (i.e., the ability for humans to maintain control and influence over AI systems and their outcomes) was emphasized, particularly in regard to critical thinking in decision-making. A central tenet throughout the GPF was that humans should not “switch off as the machines switch on.” Instead, GenAI tools should be seen as a way to enhance, and not replace, human decision-making in HTA.

### Risk-based approaches to AI use

The HTAi GPF highlighted that the risks associated with using AI tools in HTA vary widely depending on the application and context. For example, the risks of using GenAI to reformat a document are vastly different from involving GenAI tools directly in HTA decision-making (such as reimbursement recommendations) (31). Therefore, the GPF considered that risk could be considered according to the type of use – for example, operational uses such as in systematic literature reviews through to uses within the decision-making context (e.g., facilitating and informing committee deliberations). The policies/procedures would then require a risk-adequate/risk-adapted range of practices from minimal oversight (e.g., human checking of a sample of GenAI-generated output) to highly regulated or even prohibition of use depending on the application and context (e.g., there was alignment that AI should not replace committee deliberations). Such flexibility would help mitigate the risk of biases, continue to build trust, and align with existing regulatory frameworks, such as the Food and Drug Administration (FDA) guidelines (32) and the EU AI Act (25). The latter has four risk categories, namely “prohibited, high risk, limited risk, and minimal risk.”

### Defining the goal of using AI in HTA activities

GPF members considered that GenAI tools should only be used in HTA activities where the use addresses a defined challenge or need, such as improvements in operational efficiencies (33). Furthermore, if GenAI tools are to be used, then doing so should be highly likely to result in benefits, and the risks of doing so should be minimal and proportionate to the potential benefits. The implementation of GenAI in HTA activities should also be phased, starting with smaller, less complex, and repetitive tasks (the “low hanging fruit”) before expanding to applications that are higher risk with potential for more significant implications, potentially in “sandbox” environments (34). This will allow interest holders to learn, adjust, and build trust in the use of GenAI tools in HTA activities over time, but in a proactive and efficient manner (noting that, overall, GPF members felt that “the time to act is now”). This phased approach mirrors current practices within the life sciences industry, such as using GenAI for internal evidence synthesis or literature reviews, and this may facilitate smoother HTA integration. The need for all system interest holders to have an understanding, and for processes and procedures to be agile and adaptable, was emphasized. Measuring the success of the use of GenAI in HTA will likely be challenging. It could focus on how the use of GenAI enhances HTA practices, though this must be done without losing sight of health system needs. Furthermore, the equity in implementation and uptake of the use of GenAI tools around the world and across interest-holder groups should be carefully monitored to ensure inequities (e.g., through variable rates of implementation of GenAI tools) are not exacerbated (4).

### Recommendations and next steps

Alongside this journal article and information sharing with HTAi membership (including IGs and committees), the GPF members proposed the following priority next steps. These priority next steps represent overarching areas for action, synthesizing a broader range of specific initiatives and proposals debated throughout the GPF sessions and represent a possible roadmap for the responsible and strategic integration of GenAI tools into HTA activities:

1. Development of a multi-interest-holder white paper on the use of GenAI tools in HTA activities. This paper should outline some overarching principles and act as a call to action to the broader HTA community (35).
2. Development of a “GenAI Community of Practice,” leveraging membership of the HTAi IG in Real World Evidence and AI, but also all other HTAi IGs and GPF members. The GenAI Community of Practice could also go beyond the HTAi membership and include other HTA-relevant organizations and fields such as implementation science and academia. Such a community could allow networking, sharing of experiences of specific GenAI tools and use cases, and development/delivery of relevant training courses. This could bring greater consistency in approaches to the adoption of GenAI tools in HTA activities, which may expedite the implementation of these tools (albeit in an appropriate manner as previously noted). This is particularly pertinent for the life sciences industry navigating the policies and processes of multiple HTA bodies across jurisdictions.
3. We recommend that HTA bodies and policy-makers formalize risk management strategies through published guidelines and consensus-based standards, tailored to specific AI use cases, using multi-interest holder and co-creation approaches employing formal consensus methods (like Delphi panels) where appropriate. Identifying standards that GenAI tools should meet, potentially alongside questions to understand how GenAI tools have been used by others and could be adopted and/or adapted to individual contexts, will facilitate the effective monitoring of the development and implementation of these tools in HTA activities.

## Meeting considerations

This article represents a summary of discussions held at the 2025 HTAi Global Policy Forum. As such, the discussion had to be constrained to focus on issues that could be addressed and discussed in a meaningful manner within the time available. The scope of the discussions, therefore, did not go into issues surrounding the generation of evidence for HTA submissions (such as synthetic controls) and the implementation of HTA recommendations (including the use of GenAI in pricing negotiations).

While the HTAi GPF successfully convened a diverse range of attendees – including representatives from “not-for-profit” organizations (i.e., HTA bodies, payers, and health systems), “for-profit” organizations (i.e., pharmaceutical, biotech, and device companies), and patient representatives – the membership of the GPF includes membership from countries that primarily have established HTA systems. While informants from beyond the GPF membership were approached for input to the Background Paper (10) to the meeting, this is a limitation of the discussion summary, as these views were not directly present at the GPF discussions. In low- and middle-income countries (LMICs) and where there are nascent HTA bodies, the value and uses of GenAI in the HTA setting may be different and potentially more impactful; this is an avenue for further discussion and debate. Further engagement with HTA interest holders in LMICs is needed to understand context-specific policy needs and opportunities for GenAI integration.

Further, the GPF primarily comprises HTA body representatives and life science industry organizations. Patient representatives are specifically consulted during the development of the Background Paper and were invited to the meeting. However, some key interest-holder groups (such as clinicians, payers, and policy-makers) were less well represented in the discussions themselves. Again, while these views were gathered during the preparation of the meeting materials, alignment with the whole of the health sector was seen as integral as part of the next steps for the HTA community.

Furthermore, the GPF was held in a moment in time (January 2025) and was informed by a background paper that was compiled through a review of the literature and interviews with experts in the field. Noting that the field of GenAI is rapidly evolving, this means that the alignment and discussions that occurred can only reflect what was known by members at the time. Issues like data sovereignty, explainability of AI, and agentic AI were touched upon briefly, but at the time, were not at the forefront of the discussions. In addition, the environmental impact of AI was also not discussed in detail, and this has gained significant traction since the HTAi GPF took place in January 2025. Increasing awareness of AI, particularly GenAI, could indeed mean that the results from the GPF would be different if it were held again.

While this article represents the overall alignment that was reached after multiple presentations, discussions, and debates, it should be noted that the perspectives were still wide-ranging and will also evolve as the use of GenAI evolves. As noted, the time to act was felt to be now – primarily so that the HTA community is proactive rather than reactive – but that this should still involve a cautious approach informed and guided by the use of rigorous scientific principles and methods. The risk of bias in the data that GenAI tools are trained on, the possible ethical consequences of increasing use of GenAI, particularly where the data may have an underlying bias or where confidentiality, copyright, or intellectual property may be compromised, needs careful consideration, and the HTA community must pay close attention to these factors.

## Conclusions

The HTAi GPF revealed broad alignment among participants about the transformative potential of GenAI tools in HTA activities. There is potential for improving efficiency in HTA activities (i.e., speed and accuracy), which in turn could increase certainty in decision-making and enable exploration of novel use cases. While there are many challenges ahead, the HTAi GPF highlighted the importance of taking a thoughtful, cautious, and well-governed approach that gives due consideration to ethical issues, human agency, and trust-building. By starting with manageable steps and leveraging existing efforts, trust can be built, and GenAI can be integrated appropriately into HTA processes. The future of an AI-supported HTA paradigm will require continuous collaboration, strategic thinking, and a commitment to building trust through transparency and accountability.

As the implementation of GenAI tools in HTA activities begins, the HTAi GPF considered that the true potential of GenAI can only be realized when AI tools are combined with human expertise and ethical oversight. This was described as a synergistic partnership, with GenAI being used as tools by humans to create more efficient, transparent, and equitable healthcare systems, rather than as a replacement for humans. As the HTAi GPF noted, the AI contributes processing power on a scale not seen before, but the real “magic” comes from human input. Harnessing this synergistic power through collaboration and building trust with flexible yet scientifically rigorous approaches are the stepping stones of the journey to the safe, ethical, and responsible integration of AI into HTA.
